# Gut microbiome predicts cognitive function and depressive symptoms in late life

**DOI:** 10.1038/s41380-024-02551-3

**Published:** 2024-04-25

**Authors:** A. Kolobaric, C. Andreescu, E. Jašarević, C. H. Hong, H. W. Roh, J. Y. Cheong, Y. K. Kim, T. S. Shin, C. S. Kang, C. O. Kwon, S. Y. Yoon, S. W. Hong, H. J. Aizenstein, H. T. Karim, S. J. Son

**Affiliations:** 1 Center for Neuroscience, University of Pittsburgh, Pittsburgh, USA; 2 Department of Psychiatry, University of Pittsburgh School of Medicine, Pittsburgh, USA; 3 Department of Obstetrics, Gynecology and Reproductive Sciences, University of Pittsburgh School of Medicine, Pittsburgh, USA; 4 Department of Computational and Systems Biology, University of Pittsburgh School of Medicine, Pittsburgh, USA; 5 Magee-Womens Research Institute, Pittsburgh, USA; 6 Department of Psychiatry, Ajou University School of Medicine, Suwon, Republic of Korea; 7 Department of Gastroenterology, Ajou University School of Medicine, Suwon, Republic of Korea; 8 Institute of MD Healthcare Inc, Seoul, Republic of Korea; 9 Department of Bioengineering, University of Pittsburgh School of Medicine, Pittsburgh, USA

**Keywords:** Depression, Neuroscience

## Abstract

Depression in older adults with cognitive impairment increases progression to dementia. Microbiota is associated with current mood and cognition, but the extent to which it predicts future symptoms is unknown. In this work, we identified microbial features that reflect current and predict future cognitive and depressive symptoms. Clinical assessments and stool samples were collected from 268 participants with varying cognitive and depressive symptoms. Seventy participants underwent 2-year follow-up. Microbial community diversity, structure, and composition were assessed using high-resolution 16 S rRNA marker gene sequencing. We implemented linear regression to characterize the relationship between microbiome composition, current cognitive impairment, and depressive symptoms. We leveraged elastic net regression to discover features that reflect current or future cognitive function and depressive symptoms. Greater microbial community diversity associated with lower current cognition in the whole sample, and greater depression in participants not on antidepressants. Poor current cognitive function associated with lower relative abundance of *Bifidobacterium*, while greater GABA degradation associated with greater current depression severity. Future cognitive decline associated with lower cognitive function, lower relative abundance of *Intestinibacter*, lower glutamate degradation, and higher baseline histamine synthesis. Future increase in depressive symptoms associated with higher baseline depression and anxiety, lower cognitive function, diabetes, lower relative abundance of Bacteroidota, and lower glutamate degradation. Our results suggest cognitive dysfunction and depression are unique states with an overall biological effect detectable through gut microbiota. The microbiome may present a noninvasive readout and prognostic tool for cognitive and psychiatric states.

## Introduction

Over 50 million people live with dementia [1, 2], burdened with symptoms such as memory loss and inability to engage in complex cognitive functions [3–7]. Characterized as a progressive neurodegenerative condition, dementia advances from preclinical stages to mild cognitive impairment (MCI) to major neurocognitive impairment, such as Alzheimer’s Disease (AD) [8, 9]. Cognitive symptoms are often comorbid with behavioral symptoms: 30–50% of those with cognitive decline experience late-life depression [10]. Cognitive decline comorbid with depression, or even sub-diagnostic depressive symptoms, decreases quality of life and increases likelihood of progression to dementia [11–17]. Prevalence and incidence of dementia and depressive symptoms are on the rise [18, 19], with the number of impacted individuals expected to double by 2050 [20, 21]. Successful prevention or management of depressive and cognitive symptoms may improve health outcomes in late life [11, 22, 23], highlighting the urgent need to identify novel treatment and prognostic approaches for depressive symptoms and dementia [24].

Recent technological advances have enabled systems-level analysis to identify novel biomarkers for diagnostics and monitoring of treatment response in a variety of psychiatric diseases. One such novel readout emerging from these studies is the microbiome [8, 25–32]. Microbial community diversity, or alpha diversity, is the most common metric reported in the gut microbiome literature and represents a reliable indicator of overall health status [33]. Alterations in alpha diversity in either direction (i.e., increase or decrease) associate with non-comorbid depression and AD [31, 34–38]. Further, many studies identified taxa that may play a role in depression [28, 31, 36, 38–47] or AD [34, 35], while a smaller number explored functional microbiota changes in depression [45, 48], often reporting conflicting results. Nevertheless, the debate continues over the utility of the microbiome and microbial community diversity metrics as a readout of varying degrees of psychiatric disease or cognitive function.

Studies comparing gut microbiome profiles in co-occurring cognitive impairment and depressive symptoms in late life are currently lacking. This is a significant knowledge gap not only due to the high comorbidity and complex interactions between cognitive function and mood but also due to their pernicious impact and frequently detrimental outcomes [15, 24, 49, 50]. Previous studies often reported conflicting results, further highlighting the need for more research in this area. Other issues include small sample sizes, categorical approaches to psychiatric diagnosis and cognitive impairment, and a lack of consideration for important covariates (i.e., age, anxiety, antidepressant use, years of education, Body Mass Index [BMI], diabetes, hypertension) [51].

This paper examines the novel hypotheses that microbial community diversity, composition, and function may reflect current and predict future cognitive function and depressive symptoms in late life. We studied a large community clinic sample of individuals with cognitive impairment and depressive symptoms by taking a dimensional approach to both cognitive function and depressive symptoms. This approach enabled us to determine whether gut microbiome community diversity associated with current depression severity (and its dependence on antidepressant use), cognitive function, and whether these sets of factors moderated one another given the synergistic effect of comorbid cognitive decline and depressive symptoms. Finally, we identify behavioral and microbial features that reflect current or predict future cognitive function and depressive symptoms using predictive machine learning approaches.

## Methods

### Participants and study design

Participants were recruited for the Biobank Innovations for chronic Cerebrovascular disease With ALZheimer’s disease Study [52] (BICWALZS), led by the Korea Disease Control and Prevention Agency for the Korea Biobank Project. BICWALZS is an ongoing biobank platform study conducted at five universities’ memory clinics and a community geriatric mental health center to coordinate and oversee research on cognitive decline and dementia. Participants were voluntarily recruited if they visited a participating neurology or memory clinic. Some of the participants were followed annually up to 4 years. Written informed consent was obtained from all participants and caregivers. Those with current/history of a severe neurological or medical condition that would interfere with the study (e.g., Parkinson’s disease, cerebral infarction, organ failure) were excluded from the study. BICWALZS is registered in the Korean National Clinical Trial Registry (KCT0003391) and approved by Institutional Review Board (AJIRB-BMR-SUR-16-362).

At the time of this analysis, BICWALZS had recruited 713 participants from 6 sites. We included participants who provided a stool sample at baseline, *N* = 292, and a subset of those (*N* = 70/292) who completed cognitive and depressive assessment at 2-year follow-up (mean follow-up duration 23.53 ± 1.78 months). All participants were Korean. All participants in this study were recruited from two sites: a memory clinic affiliated with Ajou University Hospital and from Suwon Community Geriatric Mental Health Center.

### Assessments

All participants received comprehensive psychiatric and neuropsychological evaluations described elsewhere [52, 53]. Current diagnosis of major or minor depressive disorder was determined by a psychiatrist. Diagnosis of subjective cognitive decline (SCD) was established if no impairment was detected on the Clinical Dementia Rating (CDR) [54] and Seoul Neuropsychological Screening Battery (SNSB) [55]. MCI was diagnosed based on 0.5 CDR score and expanded Mayo Criteria on mild cognitive impairment [56]. AD was diagnosed using National Institute on Aging-Alzheimer’s Association core clinical probable AD criteria [57]. Vascular dementia was diagnosed using major vascular neurocognitive disorder criteria [58].

Current depressive symptoms were evaluated using the Korean-Version of the Montgomery-Asberg Depression Rating Scale [59] (MADRS) and Korean version of the Short form of Geriatric Depression Scale (SGDS-K) [60]. Anxiety symptoms were evaluated using the South Korean version of Beck’s Anxiety Inventory (KBAI) [61, 62]. General cognitive function was evaluated using the Mini Mental Status Examination (MMSE) [63]. Additional questionnaires included The Mini Nutritional Assessment (MNA) [64], International Physical Activity Questionnaire (IPAQ) [65], lifetime alcohol consumption (average of weekly standard drinks multiplied by years of drinking), and cigarette smoking (average of packs smoked per day multiplied by years of smoking). Participants noted their history of Diabetes Mellitus, hypertension, myocardial infarction, and cardiac ischemia. Participants completed the same measures at their 2-year follow-up.

### Microbiome data collection and preprocessing

**Fig. 1:**
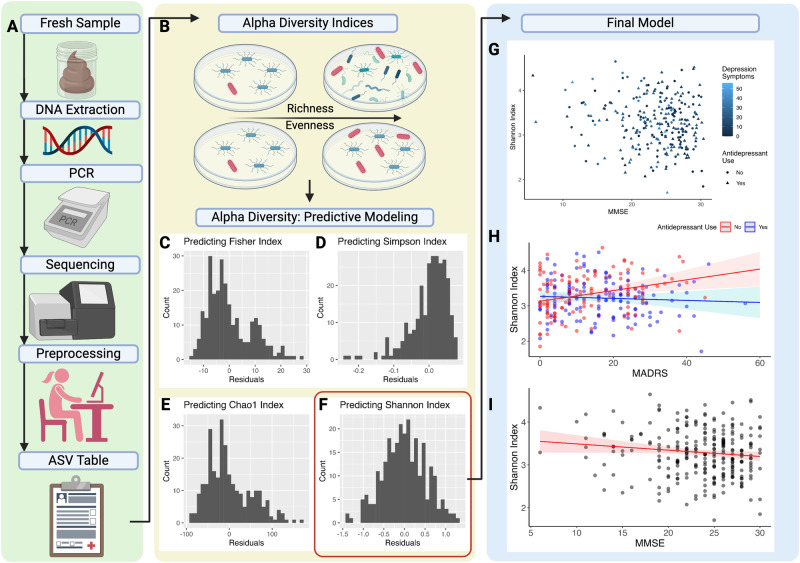
Overview of data processing and results. **A** Stool samples were collected and pre-processed to create an amplicon sequence variant table. **B** Multiple Alpha Diversity Indices were calculated to quantify gut microbiota community richness and evenness. **C**–**F** We ran four linear regression models to examine whether gut microbiome community diversity was associated with current depression severity (and its dependence on antidepressant use), cognitive function, and whether these sets of factors moderated one-another given the deleterious effect of comorbid cognitive decline and depressive symptoms. Before interpreting any of the models, we used visual inspection to ensure normally distributed model residuals. The model containing the Shannon Index as the dependent variable had the residual distribution most representative of a normal distribution, which did not violate the normality assumption. **G** Distribution of cognitive symptoms, alpha diversity, and depressive symptoms. **H** Higher microbial community diversity (alpha diversity) was associated with and greater depression severity in those participants who were not currently on antidepressants. **I** Higher microbial community diversity was associated with lower cognitive function in the whole sample. This figure was created with BioRender.com.

Stool samples were collected at the Ajou University Hospital biobank the day before clinical assessment using a sterilized stool container and stored at −20 °C until further processing (Fig. 1A). We used Illumina MiSeq platform to amplify the V3 and V4 regions of the 16 S rRNA marker gene. V3 and V4 Illumina adapters and dual barcode sequences were used to generate paired end reads of 300 bases of length in each direction.

Demultiplexed sequences were pre-processed with QIIME2 [66] (version 2022.2). First, we trimmed the primers from demultiplexed sequences. The average number of reads was 25,040 ± 6425. A trained researcher visually inspected the results to determine the read quality. Next, we denoised the data using DADA2 [67] (version 1.18), to remove chimeric sequences (sequences formed from two or more biological sequences joined together) and produce an amplicon sequence variant table. The data was truncated to minimize inclusion of poor-quality bases, while maximizing the overlap between the forward and backward reads. Taxonomy was assigned using the Silva database [68] (version 138.1). Only samples with over 10,000 reads after pre-processing were used for the subsequent analyses.

### Statistical analysis

Prior to statistical analysis, we excluded 24 participants: 15 participants were excluded for having a psychiatric disorder other than depression as their primary diagnosis (anxiety disorder: *N* = 3, sleep disorder: *N* = 1, alcohol use: *N* = 5, psychotic disorder: *N* = 3, bipolar disorder: *N* = 1, other: *N* = 2). Additional 6 participants were excluded for incomplete data, 1 for low-quality stool sample, and 2 for experimenter error. The final sample consisted of *N* = 268 participants at baseline. This cohort had 17 participants with SCD, 189 participants with MCI, 40 participants with AD, and 22 participants with another dementia (subcortical vascular dementia, AD with small vessel disease, or AD with vascular factors). Clinically, 62 participants had no psychiatric diagnosis, whereas 124 and 82 participants had a primary diagnosis of major and minor depressive disorder, respectively.

We used alpha diversity (Fig. 1B) to examine the association between high-scale gut microbiota composition, depressive symptoms, antidepressant use, and cognitive function. Alpha diversity summarizes the number and distribution of species within a community, allowing for community comparison. Alpha diversity was calculated using R library phyloseq on minimally filtered, untrimmed data to produce four indices of alpha diversity, including Fisher, Simson, Chao1, and Shannon Index. Each diversity index was used as the dependent variable in four multiple linear regression models built in R using the lm() function. All models had the same independent variables: general cognitive function (MMSE), depressive symptoms (MADRS), current antidepressant use [45] (yes or no), and an interaction factor between depressive symptoms and antidepressant use. Finally, all four models had the same covariates: participants’ age [45], sex [45], BMI [45], nutrition [69] (MNA), anxiety [70] (KBAI), exercise [71] (IPAQ), drinking [72], smoking [72], the site of testing, and presence or absence of specific medical conditions, including hypertension [73], myocardial infarction [74], cardiac ischemia [75], and diabetes mellitus [76]. The VIF function from the car package was used to assess the variance inflation factor and confirm absence of multicollinearity by ensuring that VIF < 5. Regression coefficients were standardized using lm.beta package. Processing scripts are available from the corresponding authors on reasonable request.

We used visual inspection to ensure normally distributed model residuals (Fig. 1C–F). The model containing the Shannon Index as the dependent variable had the residual distribution most representative of a normal distribution, which did not violate the normality assumption. To test for the presence of a three-way interaction between cognitive function, depressive symptoms, and antidepressant use, we built another model with Shannon Index as the dependent variable, cognitive status, depressive symptoms, antidepressant use and their interaction as the independent variable, while controlling for the same variables as previously.

Exploratory analyses were conducted to identify the predictive potential of microbial populations and microbial products in predicting cognitive functioning and depressive symptoms at baseline and 2-year follow-up. Taxonomic analyses were done on centered log-ratio transformed data agglomerated on phylum or genus level, where any unidentified taxa and any taxa occurring in less than 65% of the participants were removed (this excluded 11/15 taxa, 305/325 genus in the *N* = 268 sample, and 303/325 genus in the *N* = 70 sample). For microbial product analysis, we used PICRUSt2 [77] for functional inference in the form of Kyoto Encyclopedia of Genes and Genomes (KEGG) orthologs (KOs) [78]. We used Gut-Brain Modules (GBMs) using the Gomixer [45] library in R to extract modules corresponding to neuroactive compounds. The GBMs were restricted to a subset which may impact cognitive function [79] or depression severity [45] (Supplementary Table 1). The resulting modules were centered log-ratio transformed and any modules occurring in less than 50% of the participants were removed (this excluded 4/45 cognitive modules, and 0/19 depression modules).

We built six cross-validated elastic net models on 268 datapoints using eNetXplorer package in R to discover features predictive of baseline cognitive function and depressive symptoms. All models contained clinical and demographic controls, including participants’ age [45], sex [45], anxiety symptoms [80], years of education [81], BMI [45], use of antidepressants [45], hypertension, myocardial infarction, cardiac ischemia, and diabetes mellitus. Further, the models contained centered log-ratio transformed microbiota on phylum, genus, or functional level, and either depressive symptoms or cognitive functioning – whichever one was not the dependent variable. We used five-fold cross-validation and ran 250 permutations per model. We optimized over 50 values of lambda, and 11 values of alpha ranging from 0 to 1.

We used the same framework on 70 datapoints to predict future cognitive function and depressive symptoms at the 2-year follow-up using baseline measures. All models contained baseline information on participants’ age, sex, anxiety symptoms, years of education, BMI, use of antidepressants, cognitive function, depressive symptoms, time in months between baseline and follow-up, and log-ratio transformed microbiota phylum/genus/GBMs. As MADRS was not collected at the 2-year follow-up, we used the SGDS-K as an indicator of depressive symptoms. Both MADRS and SGDS-K are reliable scales [82, 83] and their baseline correlation in this sample was *r*(68) = 0.72, *p* < 0.0001.

## Results

Participants’ baseline clinical and demographic information is reported in Table 1. At the two-year follow-up, most participants exhibited a decrease in both cognitive function and depressive symptoms (Table 2). On average, participants experienced a 0.75 (min = −16, max = +17, SD = 4.59) reduction in MMSE and 0.41(min = −14, max = +11, SD = 3.89) reduction in GDS. Comparison of participants who agreed to versus declined to provide a stool sample is in Supplementary Table 2.

**Table 1:** Participant sample summary at baseline.

Variable	Category	Mean(SD) /*N*(%)
Sex	Female	190 (70.9)
	Male	78 (29.1)
Age		72.5 (6.9)
Education (Years)		7.6 (4.8)
BMI		23.8 (3.4)
MADRS		15.2 (11.5)
Antidepressant Use	No	136 (50.7)
	Yes	132 (49.3)
MMSE		23.4 (4.7)
KBAI		9.1 (10.1)
MNA		20.6 (4.8)
IPAQ		1126.7 (1790.2)
Lifetime Drinking (g)		10980.6 (34928.8)
Lifetime Smoking (pack-year)		7.7 (17.9)
Psychiatric Dx	None	62 (23.1)
	Major Dep	124 (46.2)
	Minor Dep	82 (30.4)
Cognitive Dx	SCD	17 (6.3)
	MCI	189 (70.5)
	AD	40 (14.9)
	Other Dementia	22 (8.2)
Hypertension	No	133 (49.6)
	Yes	135 (50.4)
Myocardial infarction	No	260 (97.0)
	Yes	8 (3.0)
Cardiac Ischemia	No	245 (91.4)
	Yes	23 (8.6)
Diabetes Mellitus	No	212 (79.1)
	Yes	56 (20.9)
Site	1	146 (54.5)
	2	122 (45.5)

*BMI* Body Mass Index, *MADRS* Montgomery-Asberg Depression Rating Scale, *MMSE* Mini Mental Status Examination, *KBAI* South Korean version of Beck’s Anxiety Inventory, *MNA* Mini Nutritional Assessment, *IPAQ* International Physical Activity Questionnaire, *Dx* Diagnosis, *SCD* Subjective Cognitive Decline, *MCI* Mild Cognitive Impairment, *AD* Alzheimer’s Disease. Site 1: Suwon Community Geriatric Mental Health Center. Site 2: Ajou University Hospital.

**Table 2:** Sample summary for *N* = 70 participants who completed 2-year follow-up.

		Baseline	Follow-up	t (df)	*P*-Value
Variable	Category	Mean(SD)/ *N*(%)	Mean(SD)		
Sex	Female	52 (74.3)			
	Male	18 (25.7)			
Age		73.2 (6.2)			
Education (Years)		7.7 (4.7)			
BMI		24.2 (4.0)			
MADRS		12.5 (9.4)			
SKGDS		6.7 (4.6)	6.3 (4.7)	1.38 (69)	0.17
Antidepressant Use	No	23 (32.9)			
	Yes	47 (67.1)			
MMSE		23.8 (5.1)	23.1 (6.3)	0.89 (69)	0.37
KBAI		6.2 (7.2)			
MNA		20.9 (3.9)			
IPAQ		1398.8 (1950.0)			
Lifetime Drinking		4289.2 (27935.6)			
Lifetime Smoking		5.2 (14.2)			
Psychiatric Dx	None	16 (22.8)			
	Major Dep	25 (35.7)			
	Minor Dep	29 (41.4)			
Cognitive Dx	SCD	4 (5.7)			
	MCI	55 (78.6)			
	AD	7 (10.0)			
	Other Dementia	4 (5.7)			
Hypertension	No	38 (54.3)			
	Yes	32 (45.7)			
Myocardial infarction	No	67 (95.7)			
	Yes	3 (4.3)			
Cardiac Ischemia	No	64 (91.4)			
	Yes	6 (8.6)			
Diabetes Mellitus	No	52 (74.3)			
	Yes	18 (25.7)			
Site	1	51 (72.9)			
	2	19 (27.1)			

*BMI* Body Mass Index, *MADRS* Montgomery-Asberg Depression Rating Scale, *SKGDS* South Korean Geriatric Depression Scale, *MMSE* Mini Mental Status Examination, *KBAI* South Korean version of Beck’s Anxiety Inventory, *MNA* Mini Nutritional Assessment, *IPAQ* International Physical Activity Questionnaire, *Dx* Diagnosis, *SCD* Subjective Cognitive Decline, *MCI* Mild Cognitive Impairment, *AD* Alzheimer’s Disease. Site 1: Suwon Community Geriatric Mental Health Center. Site 6: Ajou University Hospital. Baseline and follow-up scores were compared using a paired sample t-test.

We found that both cognitive function and depressive symptoms serve as significant predictors of microbiome composition, primarily alpha diversity, (*r* = 0.11, F(13, 254) = 2.52, *p* = 0.003, Fig. 1G–I, Table 3). Higher microbial community diversity (alpha diversity) associated with lower cognitive function in the whole sample (Fig. 1I) and greater depression severity in those participants who were not currently on antidepressants (Fig. 1H). Greater alpha diversity was also associated with lower lifetime alcohol consumption. There was no three-way-interaction between microbial community diversity, MADRS, MMSE, and antidepressant use (Supplementary Table 3).

**Table 3:** Significant predictors of alpha diversity as represented by Shannon Index.

Variable	β (SD)	Z (*p*-value)
**(Intercept)**	2.91 (0.49)	5.92***
**MMSE**	−0.13 (0.01)	−2.00*
**MADRS**	0.32 (0.01)	2.87**
Antidepressant Use [Reference Group: No]	0.13 (0.11)	1.21
MNA	0.03 (0.01)	0.34
Age	0.11 (0.00)	1.70
Sex [Reference Group: Female]	0.04 (0.10)	0.48
BMI	−0.01 (0.01)	−0.08
KBAI	0.05 (0.00)	0.61
IPAQ	−0.06 (0.00)	−0.98
Site [Reference Group: 1]	−0.15 (0.08)	−1.91
**Lifetime Drinking**	−0.19 (0.00)	−2.76**
Lifetime Smoking	0.14 (0.00)	1.95
Hypertension [Reference Group: No]	−0.04 (0.07)	−0.64
Myocardial infarction [Reference Group: No]	−0.03 (0.20)	−0.42
Cardiac Ischemia [Reference Group: No]	0.10 (0.12)	1.67
Diabetes Mellitus [Reference Group: No]	−0.11 (0.08)	−1.72
MADRS * Antidepressant Use	−0.39 (0.01)	−3.01**

β refers to standardized beta coefficients. Significance levels for *p*-values: 0.001***, 0.01**, 0.05*, 0.1. Bold variable names indicate statistical significance, *BMI* Body Mass Index. *MADRS* Montgomery-Asberg Depression Rating Scale, *MMSE* Mini Mental Status Examination, *KBAI* South Korean version of Beck’s Anxiety Inventory, *IPAQ* International Physical Activity Questionnaire, *MNA* Mini Nutritional Assessment. Site 1: Suwon Community Geriatric Mental Health Center. Site 6: Ajou University Hospital.

We leveraged machine learning to determine if microbial community metrics predict current MMSE and MADRS. Since these are data driven, exploratory analyses aimed at feature identification as opposed to hypothesis testing, we discussed all variables below *p* < 0.1 threshold. This is common when interpreting machine learning models as the goal is feature identification and the models are a combination of a complex set of weights including both significant and non-significant features especially common in microbiome literature [45].

**Fig. 2:**
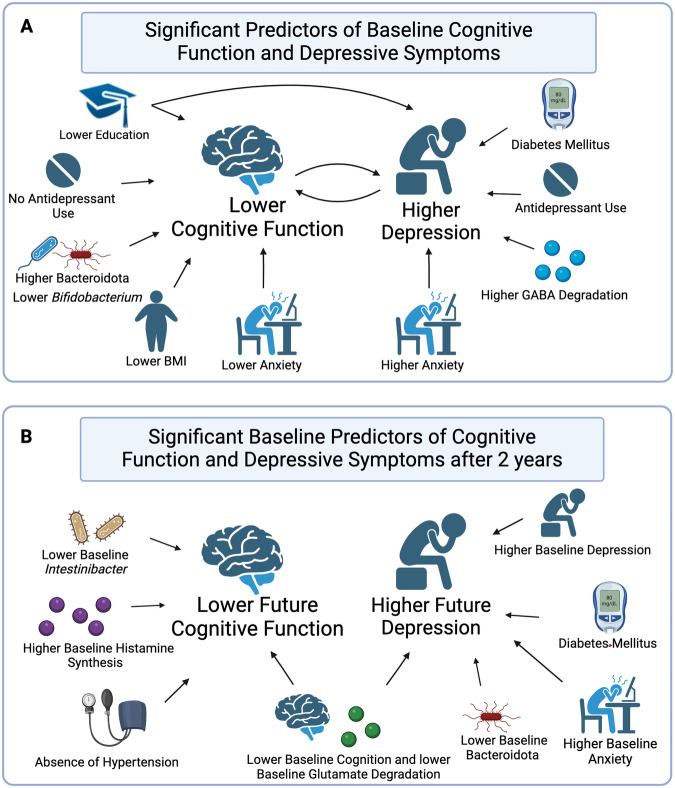
Summary of findings. **A** Lower current cognitive function is associated with lower education, no antidepressant use, higher relative levels of Bacteroidota, lower *Bifidobacterium*, lower BMI, and lower anxiety. Higher current depression is associated with antidepressant use, lower education, diabetes, higher anxiety, and higher GABA degradation. **B** Lower cognition after 2 years is associated with lower baseline cognition, lower baseline abundance of *Intestinibacter*, absence of hypertension, lower baseline glutamate degradation, and higher baseline histamine synthesis. Higher depression after 2 years is associated with higher baseline anxiety and depression, diabetes, lower baseline cognition, lower baseline Bacteroidota, and lower baseline glutamate degradation. This figure was created with BioRender.com.

Taxonomic annotation at the phylum level could accurately predict current MMSE with out-of-bag correlation coefficient r(268) = 0.42, permuted *p*-value < 0.0001, alpha=0.06, lambda = 0.0081; and with genus data with out-of-bag correlation coefficient r(268) = 0.38, permuted p-value < 0.0001, alpha=0, lambda=0.3496; and with GBM data with out-of-bag correlation coefficient r(268) = 0.35, permuted *p*-value < 0.0001, alpha=0, lambda=1.8980. These results are shown in Table 4, Fig. 2A, and Supplementary Table 4. In all models, lower MMSE (worse cognitive function) was associated with lower education and higher depressive symptoms. In the Phylum model, lower anxiety, no antidepressant use, lower BMI, and greater relative abundance of Bacteroidota associated with lower cognitive functioning. In the Genus model, lower *Bifidobacterium* abundance were associated with lower cognitive functioning. On functional level, lower propionate degradation associated with lower cognitive function.

**Table 4:** Significant baseline MMSE predictors for three separate models.

	Phylum		Genus		GBMs	
Feature	Mean β (SD)	*p*	Mean β (SD)	*p*	Mean β (SD)	*p*
Education (Years)	2.048 (0.008)	<0.001	0.799 (0.003)	<0.001	1.326 (0.006)	<0.001
MADRS	−1.104 (0.012)	0.005	−0.166 (0.014)	0.031	−0.55 (0.006)	0.006
KBAI	0.928 (0.011)	0.015				
Antidepressant Use [Reference: No]	0.565 (0.008)	0.058				
BMI	0.628 (0.01)	0.039				
Bacteroidota	−0.376 (0.033)	0.036				
Bifidobacterium					0.149 (0.007)	0.051

*Gut-Brain Modules* GBMs, *MADRS* Montgomery-Asberg Depression Rating Scale, *MMSE* Mini Mental Status Examination, *KBAI* South Korean version of Beck’s Anxiety Inventory, *BMI* Body Mass Index. All factors, including non-significant factors, are reported in Supplementary Table 4.

The results were obtained by building three separate elastic net models, one for each microbiota taxonomy/function, to predict MMSE. We optimized over 50 values of lambda and 11 values of alpha over the range [0–1]. We ran 250 permutations using five-fold cross-validation for each model. *p*-values were used to assess statistical significance of mean non-zero feature coefficients and were obtained by running a model versus null comparison. β values refer to mean of standardized regression coefficients over runs (*n* = 250), weighed by non-zero frequency over folds (*n* = 5). SD refers to standard deviation of feature coefficients over runs (*n* = 250), weighted by non-zero frequency over folds (*n* = 5).

We could predict current MADRS accurately with the phylum data with out-of-bag correlation coefficient r(268) = 0.67, permuted *p*-value < 0.0001, alpha=0.3, lambda=1.81; with the genus data with out-of-bag correlation coefficient r(268) = 0.66, permuted *p*-value < 0.0001, alpha=0.1, lambda=7.93; and with GBM data with out-of-bag correlation coefficient r(268) = 0.67, permuted *p*-value < 0.0001, alpha=0.1, lambda=7.93. Higher MADRS (greater depression severity) was associated with higher anxiety symptoms, antidepressant use, and lower cognitive functioning across all models (Table 5, Fig. 2A, Supplementary Table 5). On Phylum level, presence of Diabetes Mellitus associated with higher depressive symptoms. On Genus level, lower education associated with greater depression symptoms. On functional level, higher microbial gamma-amino butyric acid (GABA) degradation capability associated with higher depression severity.

**Table 5:** Significant baseline MADRS predictors for three separate models.

	Phylum		Genus		GBMs	
Feature	Mean β (SD)	*p*	Mean β (SD)	*p*	Mean β (SD)	*p*
KBAI	6.09 (0.014)	<0.001	4.01 (0.009)	<0.001	3.99 (0.007)	<0.001
MMSE	−1.23 (0.018)	0.007	−0.69 (0.008)	0.009	−0.71 (0.007)	0.007
Antidepressant Use [Reference: No]	0.82 (0.022)	0.047	0.6 (0.007)	0.018	0.59 (0.006)	0.02
Education (Years)			−0.39 (0.028)	0.093		
Diabetes Mellitus [Reference: No]	0.69 (0.031)	0.089				
GABA degradation					0.55 (0.015)	0.026

*Gut-Brain Modules* GBMs, *MADRS* Montgomery-Asberg Depression Rating Scale, *MMSE* Mini Mental Status Examination, *KBAI* South Korean version of Beck’s Anxiety Inventory, *GABA* γ-Aminobutyric acid. All factors, including non-significant factors, are reported in Supplementary Table 5.

The results were obtained by building three separate elastic net models, one for each microbiota taxonomy/function, to predict MADRS. We optimized over 50 values of lambda and 11 values of alpha over the range [0–1]. We ran 250 permutations using five-fold cross-validation for each model. *p*-values were used to assess statistical significance of mean non-zero feature coefficients and were obtained by running a model versus null comparison. β values refer to mean of standardized regression coefficients over runs (*n* = 250), weighed by non-zero frequency over folds (*n* = 5). SD refers to standard deviation of feature coefficients over runs (*n* = 250), weighted by non-zero frequency over folds (*n* = 5).

As our results suggest that key aspects of the microbiome, such as taxonomic annotation and alpha diversity, predict current MMSE and MADRS, we next examined if microbial features could predict cognitive and depressive outcomes at the two-year follow up visit.

We could predict future MMSE using the phylum data with out-of-bag correlation coefficient r(70) = 0.67, permuted p-value < 0.0001, alpha=0.2, lambda=0.6706; with genus data with out-of-bag correlation coefficient r(70) = 0.64, permuted p-value < 0.0001, alpha=0.1, lambda=2.1455; and with GBM data with out-of-bag correlation coefficient r(70) = 0.62, permuted *p*-value < 0.0001, alpha=0.1, lambda=2.5892 (Table 6, Fig. 2B, Supplementary Table 6). Across all models, lower MMSE at 2-year follow-up (indicating cognitive decline) was associated with lower baseline cognitive function. Cognitive decline was also associated absence of hypertension in the phylum model, lower *Intestinibacter* in the genus model, and lower Glutamate degradation and greater histamine synthesis potential in the GBM model.

**Table 6:** Significant 2-year MMSE predictors for three separate models.

	Phylum		Genus		GBMs	
Feature	Mean β (SD)	*p*	Mean β (SD)	*p*	Mean β (SD)	*p*
MMSE	3.50 (0.032)	<0.001	2.80 (0.033)	<0.001	2.57 (0.029)	<0.001
Hypertension [Reference: No]	1.01 (0.028)	0.058				
*Intestinibacter*			1.14 (0.027)	0.014		
Glutamate degradation I					−0.69 (0.024)	0.078
Histamine synthesis					0.67 (0.073)	0.092

*GBMs* Gut-Brain Modules. All predictive features were collected at baseline. *MMSE* Mini Mental Status Examination. All features, including non-significant features, are reported in Supplementary Table 6.

The results were obtained by building three separate elastic net models, one for each microbiota taxonomy/function, to predict MMSE at 2-year follow-up. We optimized over 50 values of lambda and 11 values of alpha over the range [0–1]. We ran 250 permutations using five-fold cross-validation for each model. *p*-values were used to assess statistical significance of mean non-zero feature coefficients and were obtained by running a model versus null comparison. β values refer to mean of standardized regression coefficients over runs (*n* = 250), weighed by non-zero frequency over folds (*n* = 5). SD refers to standard deviation of feature coefficients over runs (*n* = 250), weighted by non-zero frequency over folds (*n* = 5).

We could predict future SGDS-K using the phylum data with out-of-bag correlation coefficient r(70) = 0.56, permuted *p*-value < 0.0001, alpha=0.0, lambda=0.3020; with genus data with out-of-bag correlation coefficient r(70) = 0.54, permuted *p*-value < 0.0001, alpha=0.1, lambda=6.7140; and with GBM data with out-of-bag correlation coefficient r(70) = 0.52, permuted *p*-value < 0.0001, alpha=0.0, lambda=5.0645 (Table 7, Fig. 2B, Supplementary Table 7). In all three models, higher SGDS-K at 2-year follow-up (higher depression) was associated with higher baseline depression. Higher 2-year SGDS-K was also associated with lower Bacteroidota in the Phylum model and higher anxiety in the Genus model. In the GBM model, higher SGDS-K was additionally associated with greater anxiety, presence of diabetes mellitus, lower cognitive function, and lower Glutamate Degradation potential by the gut microbiota.

**Table 7:** Significant 2-year SGDS-K predictors for three separate models.

	Phylum		Genus		GBMs	
Feature	Mean β (SD)	*p*	Mean β (SD)	*p*	Mean β (SD)	*p*
SGDS-K	2.63 (0.035)	<0.001	0.89 (0.006)	<0.001	1.08 (0.008)	<0.001
KBAI			0.27 (0.01)	0.05	0.47 (0.012)	0.055
MMSE					−0.43 (0.01)	0.071
Diabetes Mellitus [Reference: No]					0.4 (0.008)	0.093
Bacteroidota	−0.58 (0.018)	0.099				
Glutamate degradation I					−0.41 (0.01)	0.08

*GBMs* Gut-Brain Modules, All predictive features were collected at baseline. *SGDS-K* South Korean short version of the Geriatric Depression Scale, *MMSE* Mini Mental Status Examination, *KBAI* South Korean version of Beck’s Anxiety Inventory. All features, including non-significant features, are reported in Supplementary Table 7.

The results were obtained by building three separate elastic net models, one for each microbiota taxonomy/function, to predict SGDS-K at 2-year follow-up. We optimized over 50 values of lambda and 11 values of alpha over the range [0–1]. We ran 250 permutations using five-fold cross-validation for each model. *p*-values were used to assess statistical significance of mean non-zero feature coefficients and were obtained by running a model versus null comparison. β values refer to mean of standardized regression coefficients over runs (*n* = 250), weighed by non-zero frequency over folds (*n* = 5). SD refers to standard deviation of feature coefficients over runs (*n* = 250), weighted by non-zero frequency over folds (*n* = 5).

## Discussion

Our analysis of a large, transdiagnostic sample of older adults illustrates novel associations between gut microbiota, cognitive function, and depressive symptoms. Greater cognitive impairment and greater depression severity (in those not on antidepressants) was associated with greater gut microbiota diversity. Our data did not support an association between the interaction of three factors and alpha diversity. In taxonomic analyses, higher abundance of Bacteroidota phylum and lower abundance of *Bifidobacterium* genus was associated with worse cognitive function at baseline. Moreover, greater gut microbial GABA degradation associated with higher baseline depression severity using an analysis of microbial metabolic pathways. Finally, we found that baseline gut microbiota predicts cognitive function and depressive symptoms at 2-year follow-up. Worse cognitive function at 2-year follow up was associated with lower baseline cognitive function and glutamate degradation, lower relative abundance of *Intestinibacter*, and higher baseline histamine synthesis. Worse depressive symptoms at 2-year follow up was associated with higher baseline depression and anxiety, diabetes, lower cognitive function, lower relative abundance of Bacteroidota and lower baseline glutamate degradation potential.

### Alpha diversity is associated with cognitive and depressive symptoms

Based on the previous literature showing associations between cognitive function and alpha diversity, we first determined whether cognitive function in an aging population was associated with gut microbiota. Lower cognitive function was associated with greater alpha diversity. These results are intriguing, given a recent meta-analysis demonstrating lower alpha diversity in those with AD compared to healthy controls [35]. It is essential to note that alpha diversity is a relative measure – high diversity is not implicitly a better or worse outcome for a community [84]. There could be multiple reasons for the differences in findings, including different patient demographics and categorical versus continuous approach to cognitive function. Nevertheless, both studies support the idea of gut dysbiosis in cognitive impairment and point toward an urgent need for a more thorough understanding of the gut-brain axis in cognitive impairment.

Based on associations between mid-life depression and gut microbiota, we investigated whether depressive symptoms in late life associated with alpha diversity. Alpha diversity was significantly associated with depressive symptoms in a way moderated by antidepressant use – greater depression severity associated with greater diversity in those not using antidepressants. These findings align with human and animal studies that implicate gut microbiota as an antidepressant response mediator [85–91]. Our findings do not align with a recent meta-analysis that did not detect alpha diversity alterations in midlife depression [92]. This may be because most previous studies lack control for covariates such as BMI and antidepressant use [30, 92, 93].

Finally, we examined whether the potential synergistic effect between comorbid cognitive decline and depressive symptoms may be reflected in the gut microbiota community composition [94]. We did not find a significant three-way interaction between cognitive function, depressive symptoms, and antidepressant use. At this time, we cannot conclude if we failed to detect the synergistic effect because it does not exist, or because it cannot be detected with the resolution offered by 16 S marker gene sequencing.

### Gut microbiome reflects current cognitive function and depressive symptoms

Next, we determined which demographic, clinical, and gut microbiome features reflect current cognitive status. We first confirmed known predictors of cognitive function, including education [81], depressive symptoms [95], anxiety [96], antidepressant use [97], and BMI [98]. We further demonstrated that a higher abundance of Bacteroidota and lower abundance of *Bifidobacterium* predict lower cognitive functioning. Bacteroidota has previously been found to associate with cognitive function in animal models [99, 100], Parkinson’s Disease [101, 102], and AD without depression [103]. We replicate and extend these findings, supporting the hypothesis that decreased abundance of Bacteroidota may be protective against dementia, potentially by reducing the amyloid load through immune-mediated pathways [104–106]. In contrast, *Bifidobacterium* is a beneficial gut genus with significant health benefits, as it suppresses inflammation and ameliorates amyloid accumulation [107, 108]. Supplementing *Bifidobacterium* improves cognitive function in animal models and people with varying levels of cognitive impairment [107–113].

Similarly, we identified features that reflect current depressive symptoms. Depression severity was associated with cognitive function [95], antidepressant use [114], anxiety symptoms [115], education level, and diabetes mellitus [116]. Higher microbial GABA degradation also associated with higher depression severity. GABA, a major inhibitory neurotransmitter, has been implicated across psychiatric disorders primarily for its role in inhibitory tuning [117]. Reduced GABAergic function is a molecular hallmark of depression [118]. Those with depression exhibit higher GABA degradation and lower GABA biosynthesis [51]. A reduction in GABAergic function plays a crucial role in cognitive function, influencing symptoms that manifest across depression and aging [119]. Microbial-derived GABA impacts GABA levels across the body and is associated with changes in behavior and functional connectivity [120]. Taken together, these findings may also point towards unique interactions between antidepressants and microbiome in psychiatric aging and drug degradation [85, 121].

### Gut microbiome predicts future cognitive function and depressive symptoms

Having established that gut microbiota is associated with current cognitive function, we next wanted to determine whether gut microbiota dynamics have predictive value for future cognitive function. Lower future cognitive function was associated with lower baseline cognitive function – a known predictor of future cognitive function [122, 123]. Surprisingly, lower future cognitive function was also associated with absence of hypertension. While some have reported a positive association between late life and hypertension and cognitive function [124], others argue that such relationship is minimal [125] or negative [126]. On taxonomic level, cognitive decline was associated with decreased relative abundance of *Intestinibacter* – a hallmark of gut dysbiosis suggesting inflammation-driven cognitive decline and increased biological aging [127–133]. Functionally, cognitive decline was associated with lower baseline glutamate degradation and higher histamine synthesis potential. The association between lower glutamate degradation potential and future cognitive decline points to the role of the gut-brain axis in glutamate excitotoxicity leading to neurodegeneration [134]. The association between increased bacterial histamine synthesis potential and future cognitive decline supports the hypothesis of neuroinflammatory-induced neurodegeneration in dementia [135].

Similarly, we confirmed features predictive of increased future depression, including higher baseline depression and anxiety, lower baseline cognitive function, and presence of diabetes mellitus. Future increase in depressive symptoms was also associated with lower relative abundance of Bacteroidota at baseline, a finding previously reported in mid-life depression [136]. These findings point to the complex interactions between depression, anxiety, aging, and cognition, further emphasizing the need to treat late-life psychiatric symptoms [137, 138]. Interestingly, higher baseline Bacteroidota associated with lower baseline cognitive function, while lower baseline Bacteroidota also associated with future depression increase, emphasizing Bacteroidota is a large phylum with a variety of species that differently impact host health. Finally, increased future depression was associated with lower baseline glutamate degradation potential. In a recent investigation, modifications in microbial metabolites preceding the metabolic processes of glutamate and GABA have been established as having a direct correlation with depression [139]. In this study, we validate and broaden the pivotal significance of microbial GABA and glutamate metabolism by elucidating that variations in both metabolic pathways are intricately linked to the manifestation of present and prospective depressive symptoms, respectively.

This study has several limitations. Our sampling was non-probabilistic, and our sample was predominately female. Our sample is inherently biased to include people with subjective or diagnosed cognitive decline. It is unclear to what extent our findings generalize to non-Korean individuals. Mood and cognition changes at the two-year-follow-up were not statistically significant, indicating that a longer follow-up period may be required. Our microbiome data was collected using 16 S rRNA marker gene sequencing and thus is not able to capture changes to gene content or microbial function. Our analysis focused on the most frequent taxa; therefore, our findings may not extend to rare ones. Extensive longitudinal studies with diverse participants sampled using shotgun sequencing are required to further elucidate the relationships between gut microbiota, cognitive impairment, and depressive symptoms in late life.

This is the first longitudinal, transdiagnostic study that investigated the current and future impacts of the gut microbiome on cognitive decline and depressive symptoms in a large sample of older adults. As such, it represents an essential step forward at the intersection of psychiatry, aging, and the microbiome. Our results suggest that the gut microbiome contributes to cognitive function and depressive symptoms across stages of cognitive impairment, whereby GABA-degrading microbiota species may be of particular interest. Further, the microbiome may predict future cognitive decline and depressive symptoms, potentially offering a biomarker for identifying people who may experience cognitive or mood decline. Such models would be of great benefit for treatment personalization which may alter disease progression and increase quality of life among the elderly.

## Supplementary information

Supplemental Table 1

Supplemental Table 2

Supplemental Table 3

Supplemental Table 4

Supplemental Table 5

Supplemental Table 6

Supplemental Table 7

## Data Availability

16 S amplicon data has been archived in NCBI SRA PRJNA 953406. Clinical data and processing scripts are available from the corresponding authors on reasonable request.
