# Supplementary material for: Gut microbiome predicts cognitive function and depressive symptoms in late life
Source: Mol Psychiatry. 2024 Apr 25;29(10):3064–75. doi: 10.1038/s41380-024-02551-3 (PMC11449789; doi:10.1038/s41380-024-02551-3)
Supplement: Supplementary file 1 — Supplemental Table 1 [file 41380_2024_2551_MOESM1_ESM.docx]

**Supplementary Table 1.**

Gut brain modules included in prediction of cognitive function and depressive symptoms.

| **Cognitive Function** | **Depressive Symptoms** | |
| --- | --- | --- |
| p-Cresol synthesis | Tryptophan degradation | |
| Inositol synthesis | Glutamate degradation I | |
| p-Cresol degradation | Glutamate degradation II | |
| Inositol degradation | Serotonin synthesis I | |
| g-Hydroxybutyric acid (GHB) degradation | Serotonin synthesis II | |
| Kynurenine degradation | Tryptophan synthesis | |
| Quinolinic acid degradation | Glutamate synthesis I | |
| Propionate synthesis III | Glutamate synthesis II | |
| Isovaleric acid synthesis I (KADH pathway) | GABA degradation | |
| Propionate degradation I | GABA synthesis I | |
| Isovaleric acid synthesis II (KADC pathway) | GABA synthesis II | |
| S-Adenosylmethionine (SAM) synthesis | GABA synthesis III | |
| Glutamate degradation II | Nitric oxide synthesis I | |
| Butyrate synthesis I | Nitric oxide synthesis II | |
| 17-beta-Estradiol degradation | Nitric oxide degradation I | |
| Butyrate synthesis II | Nitric oxide degradation II | |
| Histamine degradation | 17-beta-Estradiol degradation | |
| Quinolinic acid synthesis | Quinolinic acid synthesis | |
| Propionate synthesis II | Quinolinic acid degradation | |
| Glutamate degradation I | Isovaleric acid synthesis I | |
| GABA degradation | Isovaleric acid synthesis II | |
| Propionate synthesis I | g-Hydroxybutyric acid (GHB) degradation | |
| Nitric oxide synthesis II (nitrite reductase) | Menaquinone synthesis (vitamin K2) I | |
| Kynurenine synthesis | Menaquinone synthesis (vitamin K2) II | |
| Tryptophan synthesis |  | |
| Nitric oxide degradation I (NO dioxygenase) |  | |
| Tryptophan degradation |  | |
| Glutamate synthesis I |  | |
| Nitric oxide degradation II (NO reductase) |  | |
| Glutamate synthesis II |  | |
| ClpB (ATP-dependent chaperone protein) |  | |
| GABA synthesis III |  | |
| Acetate synthesis II |  | |
| Dopamine degradation |  | |
| Acetate synthesis III |  | |
| DOPAC synthesis |  | |
| Acetate degradation |  | |
| Nitric oxide synthesis I (NO synthase) |  | |
| Menaquinone synthesis (vitamin K2) I |  | |
| Menaquinone synthesis (vitamin K2) II (alternative pathway: futalosine pathway) |  | |
| GABA synthesis I |  | |
| GABA synthesis II |  | |
| Acetate synthesis I |  | |
| Acetylcholine synthesis |  | |
| Histamine synthesis |  |  |
