# Supplementary material for: Gut microbiome predicts cognitive function and depressive symptoms in late life
Source: Mol Psychiatry. 2024 Apr 25;29(10):3064–75. doi: 10.1038/s41380-024-02551-3 (PMC11449789; doi:10.1038/s41380-024-02551-3)
Supplement: Supplementary file 2 — Supplemental Table 2 [file 41380_2024_2551_MOESM2_ESM.docx]

**Supplementary Table 2.**

Meta data comparing participants from sites 1 and 2 who provided a stool sample versus those who did not provide a stool sample.

Note: BMI: Body Mass Index. MADRS: Montgomery-Asberg Depression Rating Scale. SKGDS: South Korean Geriatric Depression Scale. MMSE: Mini Mental Status Examination. KBAI: South Korean version of Beck’s Anxiety Inventory. MNA: Mini Nutritional Assessment. IPAQ: International Physical Activity Questionnaire. Dx: Diagnosis.

|  |  | **Site 1** | | | | | |  |
| --- | --- | --- | --- | --- | --- | --- | --- | --- |
| **Variable** | **Category** | **Missing** | **Overall** | **Did not provide a stool sample** | **Provided a stool sample** | **P-Value** | | |
| **N** |  |  | 248 | 96 | 152 |  |  |  |
| **Sex** | **Female** | 0 | 178 (71.8) | 65 (67.7) | 113 (74.3) | 0.324 |  |  |
|  | **Male** |  | 70 (28.2) | 31 (32.3) | 39 (25.7) |  |  |  |
| **Age** |  | 0 | 71.3 (7.5) | 70.5 (8.2) | 71.8 (6.9) | 0.189 |  |  |
| **Education (Years)** |  | 0 | 9.1 (4.6) | 9.0 (4.9) | 9.1 (4.4) | 0.859 |  |  |
| **BMI** |  | 0 | 24.1 (3.2) | 24.0 (3.2) | 24.1 (3.2) | 0.662 |  |  |
| **MADRS** |  | 25 | 10.6 (9.3) | 12.1 (10.2) | 9.9 (8.7) | 0.120 |  |  |
| **SKGDS** |  | 1 | 5.4 (4.5) | 5.7 (4.5) | 5.2 (4.5) | 0.460 |  |  |
| **Antidepressant Use** | **No** | 0 | 135 (54.4) | 54 (56.2) | 81 (53.3) | 0.745 |  |  |
|  | **Yes** |  | 113 (45.6) | 42 (43.8) | 71 (46.7) |  |  |  |
| **MMSE** |  | 0 | 23.5 (5.6) | 22.9 (6.2) | 23.9 (5.1) | 0.206 |  |  |
| **KBAI** |  | 0 | 4.2 (6.1) | 4.0 (6.7) | 4.4 (5.7) | 0.656 |  |  |
| **IPAQ** |  | 0 | 1098.2 (1533.2) | 1065.1 (1594.6) | 1119.1 (1498.1) | 0.791 |  |  |
| **MNA** |  | **0** | **19.8 (7.4)** | **16.3 (9.7)** | **22.0 (4.4)** | **<0.001** |  |  |
| **Lifetime Drinking** |  | 0 | 6620.8 (30115.4) | 1974.7 (7975.2) | 9555.2 (37697.9) | 0.018 |  |  |
| **Lifetime Smoking** |  | 0 | 7.7 (19.4) | 9.1 (24.4) | 6.8 (15.5) | 0.410 |  |  |
| **Cognitive Dx** | **SCD** | **0** | **25 (10.1)** | **14 (14.6)** | **11 (7.2)** | **<0.001** |  |  |
|  | **MCI** |  | **138 (55.6)** | **34 (35.4)** | **104 (68.4)** |  |  |  |
|  | **AD** |  | **57 (23.0)** | **29 (30.2)** | **28 (18.4)** |  |  |  |
|  | **ADRD** |  | **28 (11.3)** | **19 (19.8)** | **9 (5.9)** |  |  |  |
| **Psychiatric Dx** | **None** | 0 | 112 (45.2) | 49 (51.0) | 63 (41.4) | 0.632 |  |  |
|  | **Major Dep** |  | 50 (20.2) | 18 (18.8) | 32 (21.1) |  |  |  |
|  | **Minor Dep** |  | 82 (33.1) | 28 (29.2) | 54 (35.5) |  |  |  |
|  | **Bipolar disorder** |  | 2 (0.8) | 1 (1.0) | 1 (0.7) |  |  |  |
|  | **Anxiety disorder** |  | 1 (0.4) |  | 1 (0.7) |  |  |  |
|  | **Psychotic disorder** |  | 1 (0.4) |  | 1 (0.7) |  |  |  |
|  | **Other** |  | 0 |  |  |  |  |  |
| **Hypertension** | **No** | 0 | 119 (48.0) | 42 (43.8) | 77 (50.7) | 0.352 |  |  |
|  | **Yes** |  | 129 (52.0) | 54 (56.2) | 75 (49.3) |  |  |  |
| **Myocardial infarction** | **No** | 0 | 239 (96.4) | 91 (94.8) | 148 (97.4) | 0.314 |  |  |
|  | **Yes** |  | 9 (3.6) | 5 (5.2) | 4 (2.6) |  |  |  |
| **Cardiac Ischemia** | **No** | 0 | 229 (92.3) | 87 (90.6) | 142 (93.4) | 0.575 |  |  |
|  | **Yes** |  | 19 (7.7) | 9 (9.4) | 10 (6.6) |  |  |  |
| **Diabetes Mellitus** | **No** | 0 | 192 (77.4) | 75 (78.1) | 117 (77.0) | 0.956 |  |  |
|  | **Yes** |  | 56 (22.6) | 21 (21.9) | 35 (23.0) |  |  |  |

|  |  | **Site 2** | | | | | | | |  |
| --- | --- | --- | --- | --- | --- | --- | --- | --- | --- | --- |
| **Variable** | **Category** | **Missing** | **Overall** | | **Did not provide a stool sample** | | **Provided a stool sample** | | **P-Value** |  |
| **N** |  |  | 172 | | 32 | | 140 | |  |  |
| **Sex** | **Female** | 0 | 116 (67.4) | | 25 (78.1) | | 91 (65.0) | | 0.222 |  |
|  | **Male** |  | 56 (32.6) | | 7 (21.9) | | 49 (35.0) | |  |  |
| **Age** |  | 0 | 73.0 (7.0) | | 72.0 (7.9) | | 73.2 (6.7) | | 0.413 |  |
| **Education (Years)** |  | 0 | 5.9 (4.5) | | 6.2 (5.0) | | 5.8 (4.5) | | 0.700 |  |
| **BMI** |  | 0 | 23.6 (3.6) | | 23.7 (3.8) | | 23.5 (3.5) | | 0.857 |  |
| **MADRS** |  | 0 | 22.7 (12.1) | | 24.7 (10.1) | | 22.3 (12.5) | | 0.251 |  |
| **SKGDS** |  | 0 | 10.7 (4.2) | | 11.0 (4.5) | | 10.7 (4.1) | | 0.736 |  |
| **Antidepressant Use** | **No** | 0 | 84 (48.8) | | 15 (46.9) | | 69 (49.3) | | 0.960 |  |
|  | **Yes** |  | 88 (51.2) | | 17 (53.1) | | 71 (50.7) | |  |  |
| **MMSE** |  | 0 | 22.6 (4.6) | | 22.8 (4.9) | | 22.5 (4.5) | | 0.773 |  |
| **KBAI** |  | 5 | 16.1 (12.6) | | 17.6 (14.1) | | 15.8 (12.2) | | 0.501 |  |
| **IPAQ** |  | 5 | 1063.0 (1921.1) | | 620.7 (1199.4) | | 1167.9 (2044.9) | | 0.050 |  |
| **MNA** |  | 5 | 18.3 (5.4) | | 17.7 (5.3) | | 18.4 (5.4) | | 0.496 |  |
| **Lifetime Drinking** |  | 0 | 12136.6 (32214.1) | | 8470.3 (21626.7) | | 12974.6 (34183.9) | | 0.350 |  |
| **Lifetime Smoking** |  | 0 | 9.7 (21.5) | | 7.5 (17.8) | | 10.2 (22.3) | | 0.464 |  |
| **Cognitive Dx** | **SCD** | 0 | 10 (5.8) | | 2 (6.2) | | 8 (5.7) | | 0.472 |  |
|  | **MCI** |  | 124 (72.1) | | 25 (78.1) | | 99 (70.7) | |  |  |
|  | **AD** |  | 19 (11.0) | | 4 (12.5) | | 15 (10.7) | |  |  |
|  | **ADRD** |  | 19 (11.0) | | 1 (3.1) | | 18 (12.9) | |  |  |
| **Psychiatric Dx** | **Major Dep** | 0 | 119 (69.2) | | 22 (68.8) | | 97 (69.3) | | 0.774 |  |
|  | **Minor Dep** |  | 40 (23.3) | | 10 (31.2) | | 30 (21.4) | |  |  |
|  | **None** |  | 1 (0.6) | |  | | 1 (0.7) | |  |  |
|  | **Anxiety disorder** |  | 2 (1.2) | |  | | 2 (1.4) | |  |  |
|  | **Sleep disorder** |  | 1 (0.6) | |  | | 1 (0.7) | |  |  |
|  | **Alcohol use disorder** | | | 5 (2.9) | |  | | 5 (3.6) |  | |
|  | **Psychotic disorder** |  | 2 (1.2) | |  | | 2 (1.4) | |  |  |
|  | **Other** |  | 2 (1.2) | |  | | 2 (1.4) | |  |  |
| **Hypertension** | **No** | 0 | 75 (43.6) | | 11 (34.4) | | 64 (45.7) | | 0.332 |  |
|  | **Yes** |  | 97 (56.4) | | 21 (65.6) | | 76 (54.3) | |  |  |
| **Myocardial infarction** | **No** | 0 | 165 (95.9) | | 30 (93.8) | | 135 (96.4) | | 0.616 |  |
|  | **Yes** |  | 7 (4.1) | | 2 (6.2) | | 5 (3.6) | |  |  |
| **Cardiac Ischemia** | **No** | 0 | 158 (91.9) | | 31 (96.9) | | 127 (90.7) | | 0.472 |  |
|  | **Yes** |  | 14 (8.1) | | 1 (3.1) | | 13 (9.3) | |  |  |
| **Diabetes Mellitus** | **No** | 0 | 135 (78.5) | | 25 (78.1) | | 110 (78.6) | | 1.000 |  |
|  | **Yes** |  | 37 (21.5) | | 7 (21.9) | | 30 (21.4) | |  |  |
