# Supplementary material for: Gut microbiome predicts cognitive function and depressive symptoms in late life
Source: Mol Psychiatry. 2024 Apr 25;29(10):3064–75. doi: 10.1038/s41380-024-02551-3 (PMC11449789; doi:10.1038/s41380-024-02551-3)
Supplement: Supplementary file 3 — Supplemental Table 3 [file 41380_2024_2551_MOESM3_ESM.docx]

**Supplementary Table 3.**

We did not detect a significant three-way interaction between MADRS, MSSE, and antidepressant use when predicting alpha diversity, (R^2^ = 0.12, F (16, 251) = 2,11, p = 0.01). Β refers to unstandardized beta coefficients. Significance levels for p values: 0.001***, 0.01**, 0.05*. Bold variable names indicate statistical significance. BMI: Body Mass Index. MADRS: Montgomery-Asberg Depression Rating Scale. MMSE: Mini Mental Status Examination. KBAI: South Korean version of Beck’s Anxiety Inventory. IPAQ: International Physical Activity Questionnaire. MNA: Mini Nutritional Assessment. Site 1: Suwon Community Geriatric Mental Health Center. Site 6: Ajou University Hospital.

| **Variable** | **Β (SD)** | **Z (significance)** |
| --- | --- | --- |
| **(Intercept)** | **2.90 (0.62)** | **4.68 ***** |
| MMSE | -0.02 (0.02) | -1.05 |
| MADRS | 0.02 (0.02) | 0.83 |
| Antidepressant Use [Reference Group: No] | -0.38 (0.58) | -0.67 |
| MNA | 0.01 (0.01) | 0.59 |
| Age | 0.01 (0.01) | 1.73 . |
| Sex [Reference Group: Female] | 0.04 (0.10) | 0.37 |
| BMI | 0.00 (0.01) | -0.15 |
| KBAI | 0.00 (0.00) | 0.63 |
| IPAQ | 0.00 (0.00) | -0.97 |
| Site [Reference Group: 1] | -0.16 (0.08) | -1.89 . |
| **Lifetime Drinking** | **0.00 (0.00)** | **-2.64 **** |
| **Lifetime Smoking** | **0.00 (0.00)** | **2.01 *** |
| Hypertension [Reference Group: No] | -0.04 (0.07) | -0.63 |
| Myocardial infarction [Reference Group: No] | -0.12 (0.20) | -0.59 |
| Cardiac Ischemia [Reference Group: No] | 0.21 (0.12) | 1.75 . |
| Diabetes Mellitus [Reference Group: No] | -0.15 (0.09) | -1.71 . |
| MMSE * MADRS | 0.00 (0.00) | -0.09 |
| MMSE * Antidepressant Use | 0.02 (0.02) | 0.94 |
| MADRS * Antidepressant Use | 0.00 (0.03) | 0.15 |
| MMSE * MADRS * Antidepressant Use | 0.00 (0.00) | -0.77 |
|  |  |  |
|  |  |  |
| Significance levels for p values: 0.001***, 0.01**, 0.05*, 0.1·. |  |  |
