# Supplementary material for: Gut microbiome predicts cognitive function and depressive symptoms in late life
Source: Mol Psychiatry. 2024 Apr 25;29(10):3064–75. doi: 10.1038/s41380-024-02551-3 (PMC11449789; doi:10.1038/s41380-024-02551-3)
Supplement: Supplementary file 4 — Supplemental Table 4 [file 41380_2024_2551_MOESM4_ESM.docx]

**Supplementary Table 4.**

Baseline MMSE predictors for three separate models. MADRS: Montgomery-Asberg Depression Rating Scale. MMSE: Mini Mental Status Examination. KBAI: South Korean version of Beck’s Anxiety Inventory.

|  | **Phylum** | | **Genus** | | **GBMs** | |
| --- | --- | --- | --- | --- | --- | --- |
| **Feature** | **Mean β (SD)** | **p** | **Mean β (SD)** | **p** | **Mean β (SD)** | **p** |
| **madrs** | -1.104(0.012) | 0.005 | -0.166(0.014) | 0.031 | -0.55(0.006) | 0.006 |
| **Age** | 0.494(0.009) | 0.109 | 0.009(0.022) | 0.826 | 0.29(0.007) | 0.158 |
| **Sex** | 0.388(0.009) | 0.185 | 0.074(0.018) | 0.332 | 0.325(0.006) | 0.109 |
| **KBAI** | 0.928(0.011) | 0.015 | nan(0.0) | 1 | 0.267(0.006) | 0.179 |
| **Education (Years)** | 2.048(0.008) | 0 | 0.799(0.003) | 0 | 1.326(0.006) | 0 |
| **Antidep Use [Ref: No]** | 0.565(0.008) | 0.058 | 0.033(0.026) | 0.699 | 0.204(0.006) | 0.317 |
| **BMI** | 0.628(0.01) | 0.039 | 0.048(0.038) | 0.587 | 0.282(0.007) | 0.163 |
| **Hypertension** | -0.173(0.012) | 0.568 | -0.039(0.029) | 0.641 | -0.208(0.006) | 0.31 |
| **Myocardial infarction** | -0.152(0.03) | 0.614 | -0.067(0.051) | 0.43 | -0.144(0.014) | 0.479 |
| **Cardiac Ischemia** | -0.391(0.01) | 0.184 | -0.023(0.021) | 0.782 | -0.179(0.007) | 0.374 |
| **Diabetes Mellitus** | -0.2(0.013) | 0.504 | -0.03(0.022) | 0.727 | -0.222(0.006) | 0.275 |
| **Actinobacteriota** | 0.07(0.023) | 0.829 |  |  |  |  |
| **Firmicutes** | -0.058(0.018) | 0.871 |  |  |  |  |
| **Proteobacteria** | 0.118(0.046) | 0.615 |  |  |  |  |
| **Bacteroidota** | -0.376(0.033) | 0.036 |  |  |  |  |
| **Bifidobacterium** |  |  | 0.149(0.007) | 0.051 |  |  |
| **Blautia** |  |  | 0.037(0.029) | 0.652 |  |  |
| **Collinsella** |  |  | -0.046(0.031) | 0.567 |  |  |
| **Escherichia-Shigella** |  |  | 0.015(0.0) | 0.886 |  |  |
| **Streptococcus** |  |  | -0.02(0.017) | 0.836 |  |  |
| **Romboutsia** |  |  | -0.052(0.054) | 0.58 |  |  |
| **Faecalibacterium** |  |  | -0.041(0.034) | 0.642 |  |  |
| **Subdoligranulum** |  |  | -0.076(0.033) | 0.325 |  |  |
| **Anaerostipes** |  |  | 0.035(0.022) | 0.655 |  |  |
| **Erysipelotrichaceae_UCG-003** |  |  | 0.042(0.0) | 0.533 |  |  |
| **Eubacterium** |  |  | 0.035(0.027) | 0.655 |  |  |
| **Fusicatenibacter** |  |  | 0.043(0.034) | 0.604 |  |  |
| **Ruminococcus** |  |  | 0.047(0.038) | 0.586 |  |  |
| **Weissella** |  |  | 0.027(0.02) | 0.74 |  |  |
| **Ruminococcus_1** |  |  | -0.079(0.065) | 0.47 |  |  |
| **Dorea** |  |  | -0.028(0.008) | 0.731 |  |  |
| **Agathobacter** |  |  | 0.058(0.013) | 0.453 |  |  |
| **Bacteroides** |  |  | -0.026(0.026) | 0.753 |  |  |
| **Coprococcus** |  |  | -0.036(0.031) | 0.665 |  |  |
| **Eubacterium_1** |  |  | -0.046(0.03) | 0.611 |  |  |
| **p.Cresol.synthesis** |  |  |  |  | 0.137(0.006) | 0.459 |
| **Inositol.synthesis** |  |  |  |  | 0.101(0.006) | 0.611 |
| **p.Cresol.degradation** |  |  |  |  | -0.062(0.006) | 0.764 |
| **Inositol.degradation** |  |  |  |  | 0.209(0.007) | 0.264 |
| **g.Hydroxybutyric.acid..GHB..degradation** |  |  |  |  | 0.272(0.007) | 0.178 |
| **Quinolinic.acid.degradation** |  |  |  |  | 0.057(0.005) | 0.695 |
| **Propionate.synthesis.III** |  |  |  |  | -0.085(0.006) | 0.655 |
| **Isovaleric.acid.synthesis.I..KADH.pathway.** |  |  |  |  | 0.017(0.008) | 0.932 |
| **Propionate.degradation.I** |  |  |  |  | 0.318(0.006) | 0.104 |
| **Isovaleric.acid.synthesis.II..KADC.pathway.** |  |  |  |  | -0.032(0.004) | 0.781 |
| **S.Adenosylmethionine..SAM..synthesis** |  |  |  |  | 0.05(0.004) | 0.67 |
| **Glutamate.degradation.II** |  |  |  |  | 0.126(0.007) | 0.503 |
| **Butyrate.synthesis.I** |  |  |  |  | -0.131(0.006) | 0.498 |
| **X17.beta.Estradiol.degradation** |  |  |  |  | -0.069(0.006) | 0.717 |
| **Butyrate.synthesis.II** |  |  |  |  | 0.17(0.007) | 0.393 |
| **Histamine.degradation** |  |  |  |  | 0.083(0.006) | 0.651 |
| **Quinolinic.acid.synthesis** |  |  |  |  | 0.069(0.005) | 0.622 |
| **Propionate.synthesis.II** |  |  |  |  | -0.09(0.006) | 0.655 |
| **Glutamate.degradation.I** |  |  |  |  | -0.134(0.007) | 0.507 |
| **GABA.degradation** |  |  |  |  | 0.194(0.006) | 0.317 |
| **Propionate.synthesis.I** |  |  |  |  | 0.127(0.007) | 0.531 |
| **Nitric.oxide.synthesis.II..nitrite.reductase.** |  |  |  |  | 0.021(0.006) | 0.912 |
| **Tryptophan.synthesis** |  |  |  |  | 0.142(0.005) | 0.381 |
| **Nitric.oxide.degradation.I..NO.dioxygenase.** |  |  |  |  | -0.162(0.007) | 0.42 |
| **Tryptophan.degradation** |  |  |  |  | -0.13(0.007) | 0.501 |
| **Glutamate.synthesis.I** |  |  |  |  | -0.057(0.005) | 0.667 |
| **Nitric.oxide.degradation.II..NO.reductase.** |  |  |  |  | -0.142(0.006) | 0.461 |
| **Glutamate.synthesis.II** |  |  |  |  | -0.049(0.005) | 0.722 |
| **ClpB..ATP.dependent.chaperone.protein.** |  |  |  |  | 0.073(0.004) | 0.588 |
| **GABA.synthesis.III** |  |  |  |  | -0.09(0.007) | 0.648 |
| **Acetate.synthesis.II** |  |  |  |  | -0.2(0.006) | 0.313 |
| **Dopamine.degradation** |  |  |  |  | -0.226(0.006) | 0.248 |
| **Acetate.synthesis.III** |  |  |  |  | -0.153(0.006) | 0.455 |
| **DOPAC.synthesis** |  |  |  |  | -0.23(0.007) | 0.243 |
| **Acetate.degradation** |  |  |  |  | -0.084(0.008) | 0.672 |
| **Menaquinone.synthesis..vitamin.K2..I** |  |  |  |  | 0.03(0.007) | 0.875 |
| **Menaquinone.synthesis..vitamin.K2..II..**  **alternative.pathway..futalosine.pathway.** |  |  |  |  | 0.072(0.006) | 0.712 |
| **GABA.synthesis.I** |  |  |  |  | 0.198(0.006) | 0.281 |
| **GABA.synthesis.II** |  |  |  |  | -0.281(0.007) | 0.153 |
| **Acetate.synthesis.I** |  |  |  |  | 0.1(0.005) | 0.538 |
| **Histamine.synthesis** |  |  |  |  | 0.045(0.006) | 0.816 |
