# Supplementary material for: Gut microbiome predicts cognitive function and depressive symptoms in late life
Source: Mol Psychiatry. 2024 Apr 25;29(10):3064–75. doi: 10.1038/s41380-024-02551-3 (PMC11449789; doi:10.1038/s41380-024-02551-3)
Supplement: Supplementary file 5 — Supplemental Table 5 [file 41380_2024_2551_MOESM5_ESM.docx]

**Supplementary Table 5.**

Baseline MADRS predictors for three separate models. MADRS: Montgomery-Asberg Depression Rating Scale. MMSE: Mini Mental Status Examination. KBAI: South Korean version of Beck’s Anxiety Inventory. GABA: γ-Aminobutyric acid.

|  | **Phylum** | | **Genus** | | **GBMs** | | |
| --- | --- | --- | --- | --- | --- | --- | --- |
| **Feature** | **Mean β (SD)** | **p** | **Mean β (SD)** | **p** | **Mean β (SD)** | **p** |  |
| **MMSE** | -1.232(0.018) | 0.007 | -0.692(0.008) | 0.009 | -0.711(0.007) | 0.007 |  |
| **Age** | 0.132(0.115) | 0.777 | 0.008(0.115) | 0.703 | -0.0(0.088) | 0.834 |  |
| **Sex** | 0.144(0.102) | 0.757 | 0.065(0.061) | 0.809 | 0.06(0.054) | 0.822 |  |
| **KBAI** | 6.093(0.014) | 0 | 4.012(0.009) | 0 | 3.992(0.007) | 0 |  |
| **Education (Years)** | -0.319(0.09) | 0.436 | -0.391(0.028) | 0.093 | -0.364(0.032) | 0.117 |  |
| **Antidep Use [Ref: No]** | 0.823(0.022) | 0.047 | 0.6(0.007) | 0.018 | 0.589(0.006) | 0.02 |  |
| **BMI** | -0.187(0.108) | 0.669 | -0.114(0.083) | 0.666 | -0.108(0.08) | 0.687 |  |
| **Hypertension** | 0.159(0.117) | 0.728 | 0.085(0.073) | 0.757 | 0.085(0.074) | 0.757 |  |
| **Myocardial infarction** | 0.083(0.209) | 0.712 | 0.01(0.126) | 0.756 | 0.002(0.118) | 0.796 |  |
| **Cardiac Ischemia** | -0.037(0.053) | 0.923 | 0.036(0.008) | 0.886 | 0.02(0.012) | 0.947 |  |
| **Diabetes Mellitus** | 0.689(0.031) | 0.089 | 0.374(0.043) | 0.118 | 0.372(0.041) | 0.12 |  |
| **Actinobacteriota** | 0.1(0.09) | 0.816 |  |  |  |  |  |
| **Firmicutes** | -0.064(0.088) | 0.878 |  |  |  |  |  |
| **Proteobacteria** | 0.018(0.104) | 0.854 |  |  |  |  |  |
| **Bacteroidota** | -0.015(0.107) | 0.861 |  |  |  |  |  |
| **Bifidobacterium** |  |  | -0.097(0.081) | 0.709 |  |  |  |
| **Blautia** |  |  | -0.093(0.073) | 0.714 |  |  |  |
| **Collinsella** |  |  | 0.126(0.059) | 0.603 |  |  |  |
| **Escherichia-Shigella** |  |  | -0.222(0.126) | 0.383 |  |  |  |
| **Streptococcus** |  |  | 0.094(0.099) | 0.734 |  |  |  |
| **Romboutsia** |  |  | 0.193(0.053) | 0.428 |  |  |  |
| **Faecalibacterium** |  |  | -0.075(0.026) | 0.764 |  |  |  |
| **Subdoligranulum** |  |  | -0.093(0.069) | 0.709 |  |  |  |
| **Anaerostipes** |  |  | -0.071(0.045) | 0.807 |  |  |  |
| **Erysipelotrichaceae_UCG-003** |  |  | -0.085(0.072) | 0.744 |  |  |  |
| **Eubacterium** |  |  | -0.077(0.062) | 0.754 |  |  |  |
| **Fusicatenibacter** |  |  | -0.263(0.027) | 0.265 |  |  |  |
| **Ruminococcus** |  |  | -0.083(0.075) | 0.755 |  |  |  |
| **Weissella** |  |  | 0.132(0.076) | 0.603 |  |  |  |
| **Ruminococcus_1** |  |  | -0.264(0.116) | 0.401 |  |  |  |
| **Dorea** |  |  | -0.091(0.013) | 0.706 |  |  |  |
| **Agathobacter** |  |  | -0.073(0.049) | 0.769 |  |  |  |
| **Bacteroides** |  |  | -0.089(0.076) | 0.74 |  |  |  |
| **Coprococcus** |  |  | 0.059(0.056) | 0.825 |  |  |  |
| **Eubacterium_1** |  |  | -0.082(0.069) | 0.757 |  |  |  |
| **Tryptophan.degradation** |  |  |  |  | 0.094(0.08) | 0.704 |  |
| **Glutamate.degradation.I** |  |  |  |  | -0.122(0.089) | 0.628 |  |
| **Glutamate.degradation.II** |  |  |  |  | 0.047(0.039) | 0.856 |  |
| **Tryptophan.synthesis** |  |  |  |  | -0.083(0.055) | 0.674 |  |
| **Glutamate.synthesis.I** |  |  |  |  | nan(0.0) | 1 |  |
| **Glutamate.synthesis.II** |  |  |  |  | nan(0.0) | 1 |  |
| **GABA.degradation** |  |  |  |  | 0.551(0.015) | 0.026 |  |
| **GABA.synthesis.I** |  |  |  |  | -0.035(0.031) | 0.894 |  |
| **GABA.synthesis.II** |  |  |  |  | 0.051(0.048) | 0.848 |  |
| **GABA.synthesis.III** |  |  |  |  | -0.085(0.071) | 0.735 |  |
| **Nitric.oxide.synthesis.II..nitrite.reductase.** |  |  |  |  | -0.114(0.072) | 0.644 |  |
| **Nitric.oxide.degradation.II..NO.reductase.** |  |  |  |  | -0.077(0.064) | 0.76 |  |
| **X17.beta.Estradiol.degradation** |  |  |  |  | 0.075(0.062) | 0.727 |  |
| **Quinolinic.acid.synthesis** |  |  |  |  | -0.005(0.0) | 0.962 |  |
| **Quinolinic.acid.degradation** |  |  |  |  | 0.067(0.062) | 0.692 |  |
| **Isovaleric.acid.synthesis.II..KADC.pathway.** |  |  |  |  | 0.089(0.065) | 0.596 |  |
| **g.Hydroxybutyric.acid..GHB..degradation** |  |  |  |  | 0.051(0.026) | 0.872 |  |
| **Menaquinone.synthesis..vitamin.K2..I** |  |  |  |  | 0.078(0.073) | 0.744 |  |
| **Menaquinone.synthesis..vitamin.K2..II..alternative**  **pathway..futalosine.pathway.** |  |  |  |  | -0.079(0.059) | 0.77 |  |
