# Supplementary material for: Gut microbiome predicts cognitive function and depressive symptoms in late life
Source: Mol Psychiatry. 2024 Apr 25;29(10):3064–75. doi: 10.1038/s41380-024-02551-3 (PMC11449789; doi:10.1038/s41380-024-02551-3)
Supplement: Supplementary file 6 — Supplemental Table 6 [file 41380_2024_2551_MOESM6_ESM.docx]

**Supplementary Table 6.**

2-year MMSE predictors for three separate models. MMSE: Mini Mental Status Examination. KBAI: South Korean version of Beck’s Anxiety Inventor.

|  | **Phylum** | | **Genus** | | **GBMs** | |
| --- | --- | --- | --- | --- | --- | --- |
| **Feature** | **Mean β (SD)** | **p** | **Mean β (SD)** | **p** | **Mean β (SD)** | **p** |
| **gds** | 0.217(0.063) | 0.707 | 0.182(0.049) | 0.696 | 0.136(0.049) | 0.748 |
| **MMSE** | 3.502(0.032) | 0 | 2.801(0.033) | 0 | 2.57(0.029) | 0 |
| **Age** | 0.216(0.062) | 0.703 | 0.23(0.061) | 0.641 | 0.24(0.048) | 0.563 |
| **Sex** | 0.426(0.042) | 0.435 | 0.43(0.027) | 0.344 | 0.246(0.034) | 0.567 |
| **KBAI** | 0.783(0.033) | 0.137 | 0.562(0.032) | 0.217 | 0.565(0.031) | 0.164 |
| **Education (Years)** | 0.252(0.125) | 0.646 | 0.325(0.071) | 0.48 | 0.251(0.067) | 0.531 |
| **Antidep Use [Ref: No]** | 0.367(0.075) | 0.503 | 0.272(0.065) | 0.576 | 0.316(0.058) | 0.459 |
| **BMI** | -0.043(0.086) | 0.894 | 0.039(0.084) | 0.882 | 0.044(0.09) | 0.862 |
| **fu_interval_basebtwfu_month_f2** | -0.403(0.048) | 0.458 | -0.485(0.031) | 0.277 | -0.247(0.047) | 0.564 |
| **Hypertension** | 1.008(0.028) | 0.058 | 0.624(0.027) | 0.16 | 0.625(0.027) | 0.112 |
| **Myocardial infarction** | -0.022(0.079) | 0.913 | 0.107(0.082) | 0.811 | -0.023(0.039) | 0.946 |
| **Cardiac Ischemia** | -0.004(0.094) | 0.902 | -0.147(0.059) | 0.755 | 0.125(0.089) | 0.732 |
| **Diabetes Mellitus** | -0.494(0.081) | 0.347 | -0.182(0.066) | 0.703 | -0.251(0.067) | 0.55 |
| **Actinobacteriota** | -0.224(0.092) | 0.656 |  |  |  |  |
| **Firmicutes** | 0.778(0.026) | 0.116 |  |  |  |  |
| **Proteobacteria** | -0.22(0.06) | 0.635 |  |  |  |  |
| **Bacteroidota** | 0.059(0.064) | 0.896 |  |  |  |  |
| **Bifidobacterium** |  |  | -0.359(0.075) | 0.428 |  |  |
| **Blautia** |  |  | 0.202(0.084) | 0.656 |  |  |
| **Collinsella** |  |  | -0.29(0.052) | 0.539 |  |  |
| **Escherichia.Shigella** |  |  | -0.524(0.03) | 0.229 |  |  |
| **Streptococcus** |  |  | -0.154(0.055) | 0.752 |  |  |
| **Romboutsia** |  |  | -0.499(0.03) | 0.267 |  |  |
| **Faecalibacterium** |  |  | 0.135(0.076) | 0.766 |  |  |
| **Subdoligranulum** |  |  | -0.092(0.049) | 0.85 |  |  |
| **Anaerostipes** |  |  | 0.148(0.077) | 0.749 |  |  |
| **Erysipelotrichaceae_UCG.003** |  |  | -0.101(0.074) | 0.81 |  |  |
| **Eubacterium** |  |  | 0.415(0.052) | 0.355 |  |  |
| **Fusicatenibacter** |  |  | 0.343(0.072) | 0.457 |  |  |
| **Ruminococcus** |  |  | 0.4(0.045) | 0.382 |  |  |
| **Weissella** |  |  | 0.198(0.059) | 0.683 |  |  |
| **Intestinibacter** |  |  | 1.142(0.027) | 0.014 |  |  |
| **Ruminococcus_1** |  |  | 0.197(0.065) | 0.687 |  |  |
| **Dorea** |  |  | 0.13(0.068) | 0.786 |  |  |
| **Agathobacter** |  |  | 0.224(0.05) | 0.649 |  |  |
| **Bacteroides** |  |  | -0.178(0.068) | 0.678 |  |  |
| **Coprococcus** |  |  | -0.109(0.054) | 0.826 |  |  |
| **Eubacterium_1** |  |  | -0.148(0.048) | 0.765 |  |  |
| **Monoglobus** |  |  | -0.175(0.099) | 0.704 |  |  |
| **Histamine.synthesis** |  |  |  |  | -0.692(0.024) | 0.078 |
| **Glutamate.degradation.I** |  |  |  |  | 0.674(0.073) | 0.092 |
| **Inositol.degradation** |  |  |  |  | 0.512(0.035) | 0.123 |
| **GABA.synthesis.III** |  |  |  |  | -0.459(0.025) | 0.167 |
| **Histamine.degradation** |  |  |  |  | 0.306(0.036) | 0.251 |
| **GABA.synthesis.I** |  |  |  |  | 0.3(0.051) | 0.281 |
| **Tryptophan.synthesis** |  |  |  |  | -0.211(0.054) | 0.452 |
| **Glutamate.degradation.II** |  |  |  |  | 0.247(0.109) | 0.472 |
| **Nitric.oxide.degradation.II..NO.reductase.** |  |  |  |  | 0.269(0.057) | 0.492 |
| **p.Cresol.degradation** |  |  |  |  | -0.242(0.051) | 0.497 |
| **Propionate.degradation.I** |  |  |  |  | -0.234(0.044) | 0.5 |
| **Butyrate.synthesis.II** |  |  |  |  | 0.25(0.052) | 0.519 |
| **Glutamate.synthesis.II** |  |  |  |  | -0.145(0.097) | 0.578 |
| **Inositol.synthesis** |  |  |  |  | -0.188(0.073) | 0.607 |
| **Nitric.oxide.synthesis.II..nitrite.reductase.** |  |  |  |  | -0.18(0.111) | 0.639 |
| **Propionate.synthesis.I** |  |  |  |  | 0.17(0.096) | 0.645 |
| **Isovaleric.acid.synthesis.II..KADC.pathway.** |  |  |  |  | -0.089(0.065) | 0.656 |
| **ClpB..ATP.dependent.chaperone.protein.** |  |  |  |  | -0.098(0.069) | 0.657 |
| **Propionate.synthesis.II** |  |  |  |  | 0.158(0.104) | 0.672 |
| **X17.beta.Estradiol.degradation** |  |  |  |  | 0.122(0.079) | 0.696 |
| **Butyrate.synthesis.I** |  |  |  |  | 0.11(0.089) | 0.738 |
| **p.Cresol.synthesis** |  |  |  |  | -0.103(0.102) | 0.739 |
| **Propionate.synthesis.III** |  |  |  |  | -0.111(0.077) | 0.75 |
| **S.Adenosylmethionine..SAM..synthesis** |  |  |  |  | 0.063(0.06) | 0.753 |
| **Quinolinic.acid.degradation** |  |  |  |  | 0.061(0.062) | 0.76 |
| **Acetate.synthesis.III** |  |  |  |  | -0.123(0.057) | 0.761 |
| **Menaquinone.synthesis..vitamin.K2..I** |  |  |  |  | -0.104(0.08) | 0.765 |
| **Dopamine.degradation** |  |  |  |  | 0.113(0.088) | 0.768 |
| **Menaquinone.synthesis..vitamin.K2..II..**  **alternative pathway..futalosine.pathway.** |  |  |  |  | 0.127(0.068) | 0.77 |
| **Quinolinic.acid.synthesis** |  |  |  |  | 0.067(0.062) | 0.774 |
| **Glutamate.synthesis.I** |  |  |  |  | -0.056(0.046) | 0.776 |
| **DOPAC.synthesis** |  |  |  |  | -0.107(0.077) | 0.781 |
| **g.Hydroxybutyric.acid..GHB..degradation** |  |  |  |  | -0.108(0.092) | 0.782 |
| **Acetate.synthesis.II** |  |  |  |  | 0.112(0.077) | 0.786 |
| **Acetate.degradation** |  |  |  |  | -0.09(0.04) | 0.796 |
| **Acetate.synthesis.I** |  |  |  |  | -0.067(0.055) | 0.832 |
| **Tryptophan.degradation** |  |  |  |  | 0.049(0.085) | 0.834 |
| **GABA.synthesis.II** |  |  |  |  | -0.078(0.049) | 0.84 |
| **GABA.degradation** |  |  |  |  | -0.064(0.058) | 0.867 |
| **Nitric.oxide.degradation.I..NO.dioxygenase.** |  |  |  |  | -0.056(0.067) | 0.886 |
| **Isovaleric.acid.synthesis.I..KADH.pathway.** |  |  |  |  | -0.019(0.072) | 0.897 |
