# Supplementary material for: Gut microbiome predicts cognitive function and depressive symptoms in late life
Source: Mol Psychiatry. 2024 Apr 25;29(10):3064–75. doi: 10.1038/s41380-024-02551-3 (PMC11449789; doi:10.1038/s41380-024-02551-3)
Supplement: Supplementary file 7 — Supplemental Table 7 [file 41380_2024_2551_MOESM7_ESM.docx]

**Supplementary Table 7.**

2-year SGDS-K predictors for three separate models. SGDS-K: South Korean short version of the Geriatric Depression Scale. MMSE: Mini Mental Status Examination. KBAI: South Korean version of Beck’s Anxiety Inventory.

|  | **Phylum** | | **Genus** | | **GBMs** | |
| --- | --- | --- | --- | --- | --- | --- |
| **Feature** | **Mean β (SD)** | **p** | **Mean β (SD)** | **p** | **Mean β (SD)** | **p** |
| **gds** | 2.629(0.035) | 0 | 0.895(0.006) | 0 | 1.081(0.008) | 0 |
| **MMSE** | -0.819(0.036) | 0.189 | -0.2(0.021) | 0.11 | -0.429(0.01) | 0.071 |
| **Age** | 0.418(0.033) | 0.467 | 0.049(0.039) | 0.734 | 0.123(0.01) | 0.626 |
| **Sex** | -0.07(0.03) | 0.907 | -0.048(0.045) | 0.75 | -0.086(0.009) | 0.719 |
| **KBAI** | 0.628(0.039) | 0.288 | 0.268(0.01) | 0.05 | 0.468(0.012) | 0.055 |
| **Education (Years)** | 0.255(0.033) | 0.707 | -0.142(0.027) | 0.261 | -0.265(0.008) | 0.253 |
| **Antidep Use [Ref: No]** | -0.489(0.032) | 0.394 | -0.044(0.035) | 0.763 | -0.099(0.009) | 0.691 |
| **BMI** | 0.782(0.036) | 0.219 | 0.056(0.043) | 0.691 | 0.137(0.011) | 0.572 |
| **fu_interval_basebtwfu_month_f2** | 0.469(0.031) | 0.439 | -0.045(0.046) | 0.759 | -0.04(0.008) | 0.87 |
| **Hypertension** | 0.193(0.036) | 0.763 | 0.111(0.032) | 0.381 | 0.261(0.007) | 0.255 |
| **Myocardial infarction** | 0.376(0.057) | 0.528 | 0.1(0.067) | 0.411 | 0.225(0.028) | 0.398 |
| **Cardiac Ischemia** | -0.602(0.033) | 0.311 | -0.035(0.025) | 0.814 | -0.138(0.011) | 0.586 |
| **Diabetes Mellitus** | 0.256(0.031) | 0.687 | 0.16(0.024) | 0.208 | 0.4(0.008) | 0.093 |
| **Actinobacteriota** | 0.572(0.026) | 0.308 |  |  |  |  |
| **Firmicutes** | -0.322(0.028) | 0.58 |  |  |  |  |
| **Proteobacteria** | 0.452(0.019) | 0.248 |  |  |  |  |
| **Bacteroidota** | -0.585(0.018) | 0.099 |  |  |  |  |
| **Bifidobacterium** |  |  | 0.012(0.059) | 0.761 |  |  |
| **Blautia** |  |  | 0.15(0.02) | 0.233 |  |  |
| **Collinsella** |  |  | 0.052(0.042) | 0.726 |  |  |
| **Escherichia.Shigella** |  |  | 0.072(0.035) | 0.59 |  |  |
| **Streptococcus** |  |  | 0.045(0.036) | 0.752 |  |  |
| **Romboutsia** |  |  | -0.055(0.038) | 0.698 |  |  |
| **Faecalibacterium** |  |  | -0.043(0.037) | 0.769 |  |  |
| **Subdoligranulum** |  |  | -0.036(0.032) | 0.789 |  |  |
| **Anaerostipes** |  |  | 0.046(0.039) | 0.749 |  |  |
| **Erysipelotrichaceae_UCG.003** |  |  | -0.055(0.049) | 0.712 |  |  |
| **Eubacterium** |  |  | 0.013(0.0) | 0.938 |  |  |
| **Fusicatenibacter** |  |  | 0.007(0.007) | 0.96 |  |  |
| **Ruminococcus** |  |  | 0.077(0.047) | 0.565 |  |  |
| **Weissella** |  |  | -0.037(0.04) | 0.798 |  |  |
| **Intestinibacter** |  |  | -0.028(0.058) | 0.783 |  |  |
| **Ruminococcus_1** |  |  | -0.064(0.052) | 0.653 |  |  |
| **Dorea** |  |  | 0.062(0.049) | 0.652 |  |  |
| **Agathobacter** |  |  | -0.019(0.01) | 0.894 |  |  |
| **Bacteroides** |  |  | -0.053(0.04) | 0.693 |  |  |
| **Coprococcus** |  |  | 0.056(0.047) | 0.709 |  |  |
| **Eubacterium_1** |  |  | -0.042(0.041) | 0.782 |  |  |
| **Monoglobus** |  |  | -0.102(0.032) | 0.442 |  |  |
| **Tryptophan.degradation** |  |  |  |  | -0.018(0.01) | 0.936 |
| **Glutamate.degradation.I** |  |  |  |  | -0.415(0.01) | 0.08 |
| **Glutamate.degradation.II** |  |  |  |  | 0.244(0.008) | 0.287 |
| **Tryptophan.synthesis** |  |  |  |  | -0.121(0.007) | 0.431 |
| **Glutamate.synthesis.I** |  |  |  |  | -0.088(0.005) | 0.436 |
| **Glutamate.synthesis.II** |  |  |  |  | -0.14(0.005) | 0.28 |
| **GABA.degradation** |  |  |  |  | -0.108(0.009) | 0.64 |
| **GABA.synthesis.I** |  |  |  |  | 0.013(0.008) | 0.955 |
| **GABA.synthesis.II** |  |  |  |  | 0.096(0.008) | 0.686 |
| **GABA.synthesis.III** |  |  |  |  | -0.202(0.01) | 0.331 |
| **Nitric.oxide.synthesis.II..nitrite.reductase.** |  |  |  |  | 0.179(0.008) | 0.427 |
| **Nitric.oxide.degradation.II..NO.reductase.** |  |  |  |  | 0.119(0.007) | 0.57 |
| **X17.beta.Estradiol.degradation** |  |  |  |  | -0.206(0.006) | 0.262 |
| **Quinolinic.acid.synthesis** |  |  |  |  | 0.032(0.005) | 0.772 |
| **Quinolinic.acid.degradation** |  |  |  |  | 0.016(0.004) | 0.883 |
| **Isovaleric.acid.synthesis.II..KADC.pathway.** |  |  |  |  | 0.144(0.005) | 0.235 |
| **g.Hydroxybutyric.acid..GHB..degradation** |  |  |  |  | 0.279(0.009) | 0.236 |
| **Menaquinone.synthesis..vitamin.K2..I** |  |  |  |  | -0.297(0.007) | 0.121 |
| **Menaquinone.synthesis..vitamin.K2..II**  **alternative.pathway..futalosine.pathway.** |  |  |  |  | -0.081(0.007) | 0.731 |
